# Transformer-based molecular fragment prediction using SMILES and DeepSMILES representations in a fragment-based drug discovery pipeline

**DOI:** 10.1186/s13321-026-01255-w

**Published:** 2026-07-31

**Authors:** Aayush Kothari, Amish Gupta, Nisarg Shah, Thomas Reed, Anvita Nattuva, Rahima Nazirudeen, Harman Brah, Marx Akl

**Affiliations:** 1 Department of Computer Science & Engineering, Aspiring Scholars Directed Research Program, Fremont, CA 94539 USA; 2 Department of Chemistry, Biochemistry, and Physical Science, Aspiring Scholars Directed Research Program, Fremont, CA 94539 USA

**Keywords:** SMILES, DeepSMILES, Transformers, ChemBERTa, Tanimoto similarity, Masked language modeling, Drug design, Fragment-based drug discovery

## Abstract

Fragment-based drug discovery (FBDD) uses small molecular fragments as starting points for drug development, and machine-learning models that operate on molecular string representations are increasingly applied to fragment-related tasks. SMILES is the dominant such representation, but its paired ring closures and balanced parentheses introduce syntactic complexity that can affect model behavior. We present a controlled study of how molecular string representation influences transformer-based fragment recovery, using an FBDD-motivated label pipeline: reference fragments are derived from known drugs via RECAP fragmentation and docking-based ranking, and the model is scored on recovering them. We introduce DeepBERTa, a ChemBERTa-derived transformer pretrained on DeepSMILES, and evaluate it against a matched SMILES baseline under identical architecture, data splits, and optimization (approximately 34,000 drug–fragment pairs). DeepSMILES produces syntactically valid predictions more often than SMILES (54.2% vs. 43.5%) and a higher full test-set mean Tanimoto similarity (0.36 vs. 0.29). A per-sample selection between the two representations raises mean Tanimoto to 0.43, and a deployable variant that selects on model confidence rather than the reference recovers most of this gain. Among molecules where one representation strictly wins, DeepSMILES wins more often than SMILES (27.8% vs. 16.3% of test molecules). These results show that string representation meaningfully influences transformer fragment recovery and that representation-aware selection outperforms either representation alone.

**Supplementary Information:**

The online version contains supplementary material available at https://doi.org/10.1186/s13321-026-01255-w.

## Scientific contribution

To the best of our knowledge, this is the first systematic comparison of SMILES and DeepSMILES under matched conditions for transformer-based molecular fragment recovery. We introduce DeepBERTa, a ChemBERTa-derived transformer pretrained on DeepSMILES, and show that string representation measurably affects both syntactic validity and recovery similarity.

**Supplementary Information:**

The online version contains supplementary material available at https://doi.org/10.1186/s13321-026-01255-w.

## Introduction

**Fig. 1:**
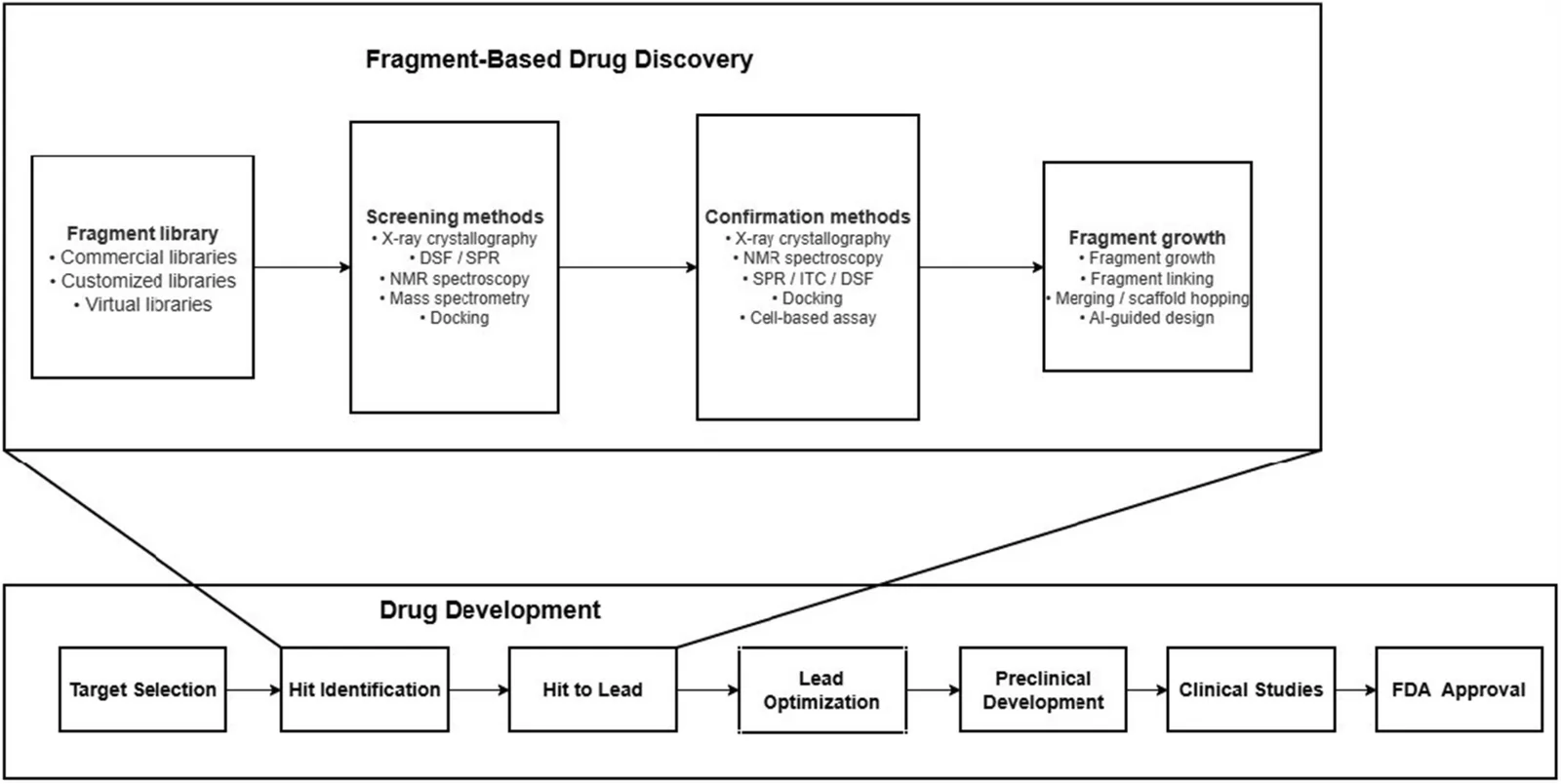
Overview of the Fragment-Based Drug Discovery (FBDD) workflow in relation to the broader Drug Development pipeline

Fragment-based drug discovery (FBDD) is a paradigm in which small molecular fragments (typically < 300 Da) are used as starting points for drug development [1], as shown in Fig. 1. Unlike high-throughput screening (HTS) of large drug-like molecules, FBDD involves screening a relatively small library of fragments to identify weak binders, which are then optimized or linked to yield lead compounds. This approach has gained traction for targets that challenge conventional screening approaches, as fragment hits can be grown or linked to produce high-affinity inhibitors, enabling more effective exploration of the chemical space [2]. Recent advances in artificial intelligence are accelerating FBDD by enabling *in silico* prediction of fragment binding and automated design of fragment-derived candidates [3, 4]. In particular, machine learning models that operate on chemical representations can help identify which substructures of a complex molecule are critical for activity, guiding medicinal chemists in fragmentation and elaboration [5].

One of the most widely used representations for encoding molecular structures is SMILES (Simplified Molecular Input Line Entry System). This linear notation treats molecules as sequences of characters, while critically retaining essential stereochemical information [6]. SMILES is supported by numerous programming libraries and integrated into widely used molecular databases such as PubChem and ZINC [7, 8]. This linear chemical language has allowed natural language processing (NLP) models to be applied to chemistry [9], where they have shown great promise for molecular property prediction and generation tasks [10–12].

One notable example is ChemBERTa, a RoBERTa-based transformer model explicitly fine-tuned for SMILES representations in chemical applications [9]. Transformer models are particularly suitable for processing such chemical representations due to their self-attention mechanism [13], which captures both local bonding patterns and long-range dependencies across molecular substructures, even when relevant atoms are separated by multiple tokens [11]. This combination enables transformers to learn robust and context-aware representations, which are vital for accuracy in molecular tasks [9].

Multiple state-of-the-art transformer models have been developed for both masked language modeling (MLM) and multitask regression (MTR) paradigms and have achieved strong performance on molecular benchmarks such as MoleculeNet [14, 15]. In addition to ChemBERTa, other notable transformer models include MolBERT [16], SMILES-BERT [11], and MolGPT [17], each pretrained on increasingly larger dataset sizes. For example, MolBERT was trained on approximately 1.6 million compounds [16], SMILES-BERT on 18.7 million compounds [11], and the latest ChemBERTa-2 on 77 million compounds [14]. More recent efforts, such as MoLFormer [18] and ChemBERTa-3 [19], have scaled pretraining to more than a billion molecules, combining PubChem [7] and ZINC [8]. This progression reflects a broader trend in molecular deep learning: increasing pretraining size leads to better generalization and chemical understanding [20].

Despite these advances, the universal adoption of SMILES has significant drawbacks. Although SMILES-based methods have been more widely adopted and tested than other chemical representations, they still face challenges in downstream tasks [21]. Furthermore, the use of paired ring closure digits and nested brackets makes SMILES strings prone to parsing errors during molecule prediction paradigms [22]. SMILES strings, while representative, can be slow to process and consume valuable computational resources [23]. These challenges lead to lower performance during molecule generation tasks by increasing syntactic errors and meaningless outputs [23].

Our work builds on the ChemBERTa paradigm of MLM on SMILES, but uses DeepSMILES as an alternative encoding to investigate whether addressing SMILES syntax issues can lead to better outcomes. DeepSMILES, introduced by O’Boyle and Dalke in 2018 [24], uses a single ring closure number instead of a pair and replaces balanced parentheses with multiple closing parentheses to indicate branch length. For example, a branch of length N is closed by N right parentheses in a row, rather than matching each opening parenthesis individually. This postfix notation has been shown to increase validity during generation and enhance computational speed [24]. Although multiple studies have explored the benefits of DeepSMILES in recurrent neural networks (RNNs) [24] and variational autoencoders (VAEs) [25], limited analysis has been done on the performance of DeepSMILES on transformers.

In this study, we examine how SMILES and DeepSMILES influence transformer-based fragment prediction under controlled conditions. We introduce DeepBERTa, a ChemBERTa-derived transformer trained on DeepSMILES, and evaluate it alongside a SMILES-based ChemBERTa model using identical architectures, data splits, and optimization. FBDD is used as a motivating setting, with fragment prediction defined as the recovery of docking-ranked retrosynthetic fragments derived from known molecules. We emphasize at the outset that this is a controlled representation-learning study using an FBDD-motivated label-generation pipeline; recovering a docking-ranked RECAP leaf fragment of a known molecule is a surrogate for fragment prioritization, not a deployable substitute for prospective fragment screening.

### Fragment-based molecular design

There is a rich history of computational methods that utilize molecular fragments, aligning with the principles of FBDD [26]. Traditional methods include algorithms like RECAP for fragmenting molecules [27] and fragment-linking strategies [28], while recent AI models incorporate fragments to improve generation. For instance, junction-tree neural networks construct molecules by joining fragment subgraphs, ensuring chemical validity by design [5]. In sequence-based models, *MolMiner*, an auto-regressive transformer, generates molecules as sequences of fragment building steps (“molecular stories”). By adding fragments one by one and applying chemical rules at each step, their approach guarantees that intermediate and final molecules are valid and enhances interpretability, since each generation step corresponds to a meaningful modification [29]. This trend of injecting domain knowledge (fragment libraries or chemical rules) into generative models addresses challenges of validity and transparency. Our work shares a similar spirit: rather than assembling molecules fragment by fragment, we focus on fragment prediction using a molecular language model.

### Robust molecular string representations

SMILES is not the only representation for string-based molecule encoding. SELFIES (Self-Referencing Embedded Strings) is another representation that guarantees 100% syntactic validity—every SELFIES string maps to a valid molecule [22]. SELFIES employs a grammar of tokens that encode chemical valence rules, allowing even random token sequences to produce valid structures. However, the ChemBERTa team found that SELFIES did not yield significant improvements, and that training with SELFIES was substantially slower while offering minimal gain. This inefficiency is a result of SELFIES producing longer token sequences and requiring larger vocabularies, which increases training time and memory consumption [9].

DeepSMILES is a recent alternative to SMILES. It does not guarantee the validity of every string, but it simplifies the syntax to reduce parsing errors and increase processing speeds [24]. Designed to address the issue of paired symbols, DeepSMILES uses a number to indicate the ring size, reducing the complexity and length of the string. Because DeepSMILES was designed with machine-learning compatibility in mind, we selected DeepSMILES over SELFIES for this study to examine representation-level differences under transformer-based modeling. Indeed, prior studies have reported differences in behavior between DeepSMILES- and SMILES-based models in generative tasks [25, 30, 31]. Examples of how representative fragments are encoded in SMILES and DeepSMILES are shown in Table 1.

We focus on DeepSMILES rather than SELFIES because SELFIES’ core advantage – guaranteed validity – does not address our task’s bottleneck. We score predictions by structural similarity to a specific reference fragment, where a valid but incorrect molecule is penalized as heavily as an invalid one, and our invalid predictions are mostly near-misses rather than syntactic noise. Recent work further suggests that enforcing validity can impair a chemical language model’s learning [32], and that the best representation is strongly task-dependent rather than universal [33]. DeepSMILES also preserves SMILES-like tokenization, keeping the comparison to our SMILES baseline controlled. A direct SELFIES baseline remains a natural extension.

**Table 1:** Fragment representations in SMILES and DeepSMILES formats

Formula	SMILES	DeepSMILES
$C_8H_9Br$	CCc1cccc(Br)c1	CCcccccBr)c6
$C_{11}H_{17}NO$	CCOC(CN)Cc1ccc(C)cc1	CCOCCN))CccccC)cc6
$C_{8}H_{8}F_2S$	Cc1cc(F)c(S(C)(=O)=O)cc1F	CcccF)cSC)=O)=O))cc6F
$C_{8}H_{10}$	Cc1ccc(C)cc1	CccccC)cc6
$C_9H_{12}O$	COc1cc(C)ccc1C	COcccC)ccc6C
$C_9H_{12}$	Cc1ccc(C)c(C)c1	CccccC)cC)c6
$C_{11}H_{10}N_2OCl$	Cn1c(=O)[nH]c2cc(Cl)ccc21	Cnc=O)[nH]cccCl)ccc69

## Methods

### Data collection and processing

Approximately 34,000 drug molecules were selected and fragmented, with fragments subsequently ranked using a docking-based scoring procedure against the mTOR protein kinase. We chose mTOR as the target for this study because of its pharmacological importance and the atypical scaffolds of its known inhibitors (e.g., the macrocyclic rapamycin), making it a challenging and illustrative test case. To generate the dataset, we first fragmented all drugs using the RECAP fragmentation algorithm [27]. We then assigned each fragment a numerical score according to a heuristic scoring function, and for every drug, selected the highest-scoring fragment and wrote the corresponding drug–fragment pair to the output training dataset. If all fragments received the lowest possible score, no pair was written for that drug. Our scoring function uses configurable weights and minimum/maximum bounds for various criteria, meant to reflect differing opinions regarding what constitutes a promising fragment candidate, as well as basic filtering. The scoring function evaluates each fragment according to the following rules:

1. *Hard filtering (automatic rejection).* A fragment is immediately assigned the lowest possible score if any of the following conditions are met:
  - It is “trivial,” defined as having fewer than 5 non-hydrogen atoms, or having fewer than 8 non-hydrogen atoms and containing only carbon and hydrogen.
  - Its molecular weight exceeds 300 Da, or its predicted logP exceeds 3 (Rule of Three) [34].
  - Its predicted logS (ESOL) [35] is less than -3.
2. *Score initialization.* If the fragment passes all hard filters, it is assigned an initial score of 0.
3. *Soft penalties and adjustments.*
  - The Rule of Three suggests that good fragments contain no more than three hydrogen bond donors and no more than three hydrogen bond acceptors (Lipinski definitions) [34]. Therefore, the score is decreased by 1 for each donor or acceptor individually exceeding three.
  - The fragment’s synthetic accessibility (SAscore) [36] is multiplied by 0.5 and subtracted from the score, penalizing synthetically complex fragments.
4. *Docking-based contribution.* The fragment is docked against the target’s active site to estimate its binding affinity and ligand efficiency (binding affinity normalized by the number of non-hydrogen atoms). This contribution is added to the fragment’s score.

To ensure reproducibility and promote future development, we made all code and datasets publicly available. In this work, “fragment prediction” refers to generating a chemically meaningful fragment that resembles the reference fragment obtained from RECAP and docking. Our comparisons evaluate structural similarity, not fragment-contribution analysis or identification of which substructures drive binding affinity.

### Overall pipeline

**Fig. 2:**
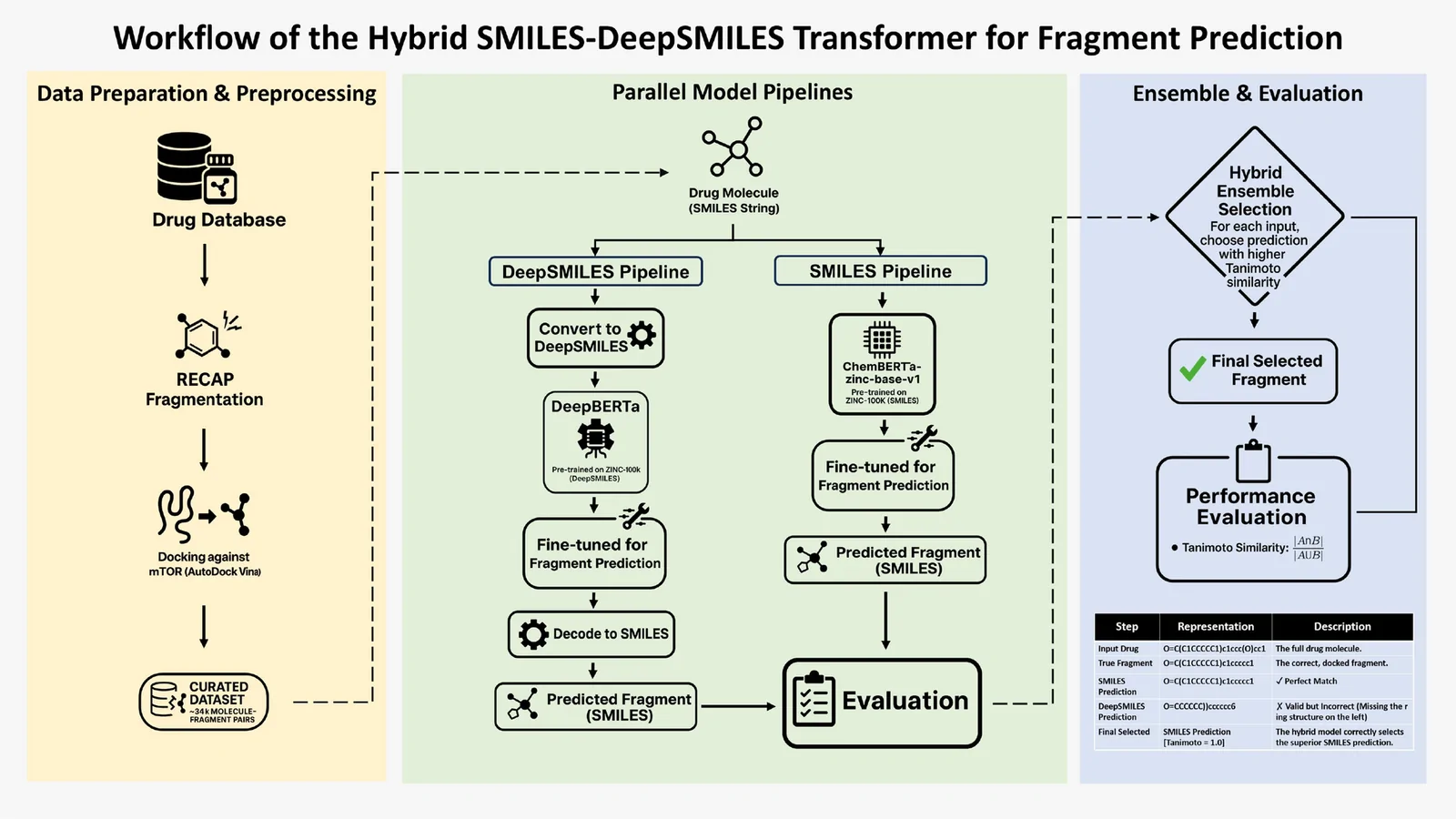
Overview of the SMILES and DeepSMILES fragment prediction pipeline. The model is trained using both SMILES and DeepSMILES molecules and predicts fragments that are decoded back to SMILES for evaluation

Our objective was to examine how SMILES and DeepSMILES representations influence transformer-based fragment prediction and to assess whether a simple hybrid selection strategy reveals complementary behavior between the two molecular encodings. To this end, we developed two parallel modeling pipelines—one based on ChemBERTa, trained on SMILES strings, and the other based on DeepBERTa, trained on DeepSMILES representations—as illustrated in Fig. 2. Each pipeline begins with pretraining on the ZINC molecular database, followed by RECAP-based fragmentation of the mTOR dataset to generate paired drug–fragment data. The resulting fragment prediction models independently output candidate fragments, which are then compared and validated. In the final stage, both outputs are evaluated, and the one with the higher Tanimoto similarity is chosen.

For the SMILES-based model, we experimented with several publicly available ChemBERTa checkpoints and found that the zinc-base-100k-v1 model hosted on Hugging Face provided the best performance in terms of fragment prediction. Even though other models were trained on larger datasets (ChemBERTa-77M-MLM), the distribution of molecules in ZINC was similar to that in our target domain [8]. After training, the predicted fragments were assessed via Tanimoto similarity to the true fragments and processed further. Post-processing was outside the scope of this study, as our goal was to evaluate raw model predictions.

The DeepSMILES-based pipeline mirrored the SMILES pipeline, but required additional pretraining from scratch. Building on the insight that zinc-base-100k-v1 performed best for SMILES, we adopted the same 100k molecule ZINC subset for pretraining. Notably, all molecules were successfully converted to DeepSMILES [8]. Using an identical RoBERTa-base architecture and the open-source ChemBERTa training framework, we trained a DeepSMILES model (DeepBERTa) that closely matches the SMILES model architecture, preserving the same number of attention heads, transformer layers, vocabulary size, and tokenizer-related parameters.

### Model architecture

**Table 2:** Comparison of model and training hyperparameters between ChemBERTa-zinc-base-v1 and DeepBERTa

Hyperparameter	ChemBERTa-v1	DeepBERTa
*Model Architecture*		
Model Type	RoBERTa-base	RoBERTa-base
Hidden Layers	6	6
Hidden Size	768	768
Intermediate Size	3072	3072
Attention Heads	12	12
Max Pos Embeddings	514	514
Hidden Activation	GELU	GELU
Attention Dropout	0.1	0.1
Hidden Dropout	0.1	0.1
Initializer Range	0.02	0.02
*Tokenizer and IDs*		
Tokenizer Type	BPE	BPE
Vocab Size	767	767
Pad Token ID	1	1
BOS Token ID	0	0
EOS Token ID	2	2
Masking Probability	0.15	0.15
*Training and Optimization*		
Optimizer	AdamW	AdamW
Epochs	∼5	∼4.2
Loss Function	MLM	MLM
*Data and Hardware*		
Training Dataset	ZINC-100k	ZINC-100k
Hardware	V100 GPU	Tesla P100

Although the DeepSMILES-based model was trained using the ChemBERTa framework [9], certain differences arose when compared with the original ChemBERTa model. Due to the partial availability of original hyperparameters, we approximated certain values [37]. We list the key hyperparameters and their values in Table 2. Nevertheless, our model—designed to imitate *ChemBERTa-zinc-base-100k-v1* (henceforth referred to as DeepBERTa)—remains architecturally comparable, preserving the same number of attention heads, transformer layers, vocabulary size, and tokenizer-related parameters.

### Training and fine-tuning strategy

We fine-tuned the ChemBERTa and DeepBERTa models on a fragment prediction task, where each training example consisted of a drug molecule (as SMILES or DeepSMILES) as input and its corresponding fragment string as the target output.

To ensure that both SMILES and DeepSMILES models were trained and evaluated on the same data, we split the dataset, which consisted of approximately 34,000 molecules, into training, validation, and test sets. The same random train-val-test split (80–15–5) was used for both SMILES and DeepSMILES to ensure comparability. We verified that all examples were distinct and that no data leakage occurred between the training, validation, and test sets. The test set contained 1,689 examples. Fragment predictions were evaluated using Tanimoto similarity [38], a widely used metric that measures structural overlap between two molecules. We utilized Morgan fingerprints [39] with a radius of 2 and a length of 2048 bits.

We trained our models using the AdamW optimizer [40] with weight decay regularization. To curb early training instability, we applied a linear learning-rate scheduler with warmup [41] over 10% of the total training steps. Furthermore, because Tanimoto similarity is not directly optimized by the model’s cross-entropy (CE) loss, we incorporated a sequence penalty term that scales the CE loss based on the predicted sequence’s Tanimoto similarity, encouraging the model to produce outputs closer to the reference fragments.

To discourage the model from producing syntactically invalid predictions, we added a penalty that scales the per-batch loss according to the fraction of invalid predictions. For a batch size of *B*, we compute

$$f_{\text {invalid}} = \frac{1}{B}\sum _{n=1}^{B}\bigl (1 - v_n\bigr ), \quad v_n = {\left\{ \begin{array}{ll} 1, & \text {if prediction }n\text { is a valid SMILES},\\ 0, & \text {otherwise}, \end{array}\right. }$$

where $v_n$ is either 0 or 1 if it passes or fails an RDKit [42] validity check. Then, we multiply it by our CE loss and regularization term to get the final loss function.

$$\mathcal {L}_{\textrm{batch}} = \Bigl (\lambda _{\textrm{seq}}\,\mathcal {L}_{\textrm{CE}} \;+\;\lambda _{\textrm{L1}}\!\sum _{j\in \theta }|\theta _j|\Bigr ) \times \bigl (1 + \alpha _{\text {invalid}}\,f_{\text {invalid}}\bigr ).$$

where $\alpha _{\text {invalid}}$ represents the weight applied to $f_{\text {invalid}}$ after being scaled by the batch size. When a specific batch contains a large number of invalid outputs, this loss is amplified. During backpropagation, this yields larger gradient updates, steering the model to reduce invalid outputs. Since any invalid output automatically yields a Tanimoto similarity of 0, we included L1 regularization with the CE loss before scaling, penalizing batches with high proportions of invalid predictions. Throughout the training process, we aimed for the model to learn to prioritize syntactically valid SMILES and DeepSMILES.

Hyperparameters, including maximum sequence length, batch size, learning rate, weight decay, and penalty coefficients, were tuned using Optuna [43] with a Tree-structured Parzen Estimator (TPE) sampler and median pruning. The best configuration was selected based on the mean Tanimoto similarity over 40 optimization trials.

Furthermore, we performed statistical comparisons of per-sample Tanimoto similarities across representations using a two-tailed Wilcoxon signed-rank test [44].

### Use of large language models

ChatGPT 5.0 and Claude Sonnet 4.6 were used only for formatting support (e.g., LaTeX tables, figure captions, and minor wording adjustments). They were not used to generate, analyze, or interpret scientific content. All material was reviewed and verified by the authors.

## Results

For each test example, we computed the Tanimoto similarity between the predicted and reference fragment: if a predicted string was invalid (could not be parsed into a molecule), it was assigned a Tanimoto similarity of 0. We also calculated the average string similarity for the best-performing models using the Sequence Matcher Library [45].

**Fig. 3:**
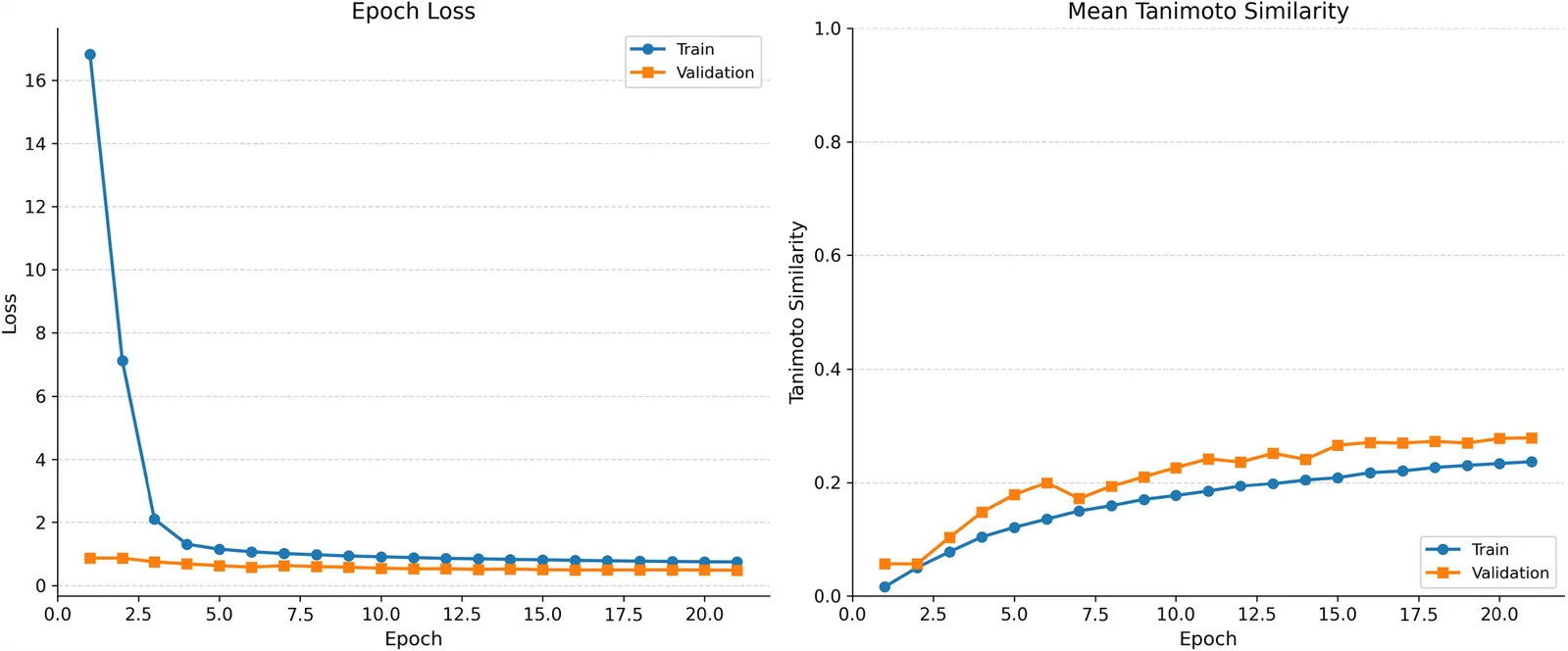
Loss (left) and mean Tanimoto similarity (right) for the best-performing SMILES-based model

**Fig. 4:**
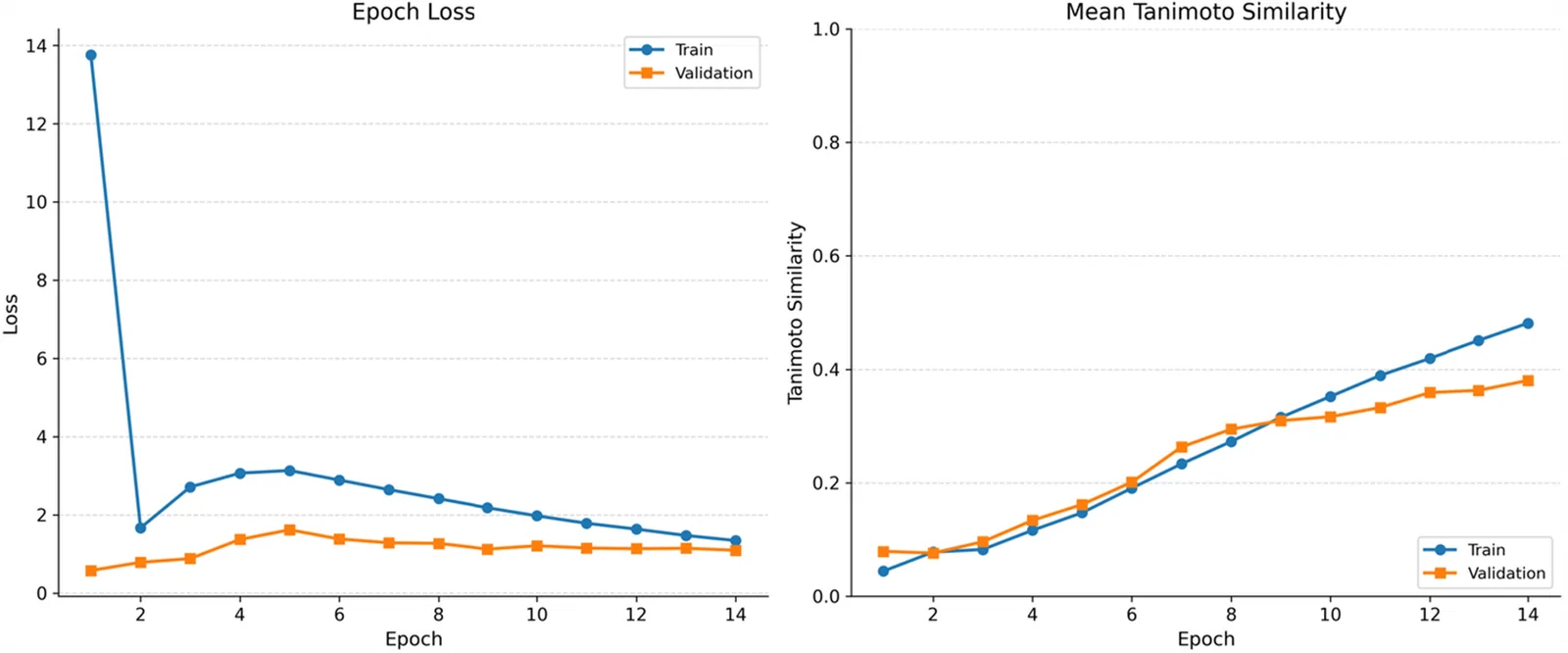
Loss (left) and mean Tanimoto similarity (right) for the best-performing DeepSMILES-based model

The loss and Tanimoto similarity plots for representative SMILES and DeepSMILES based models are shown in Figs. 3 and 4, respectively. Differences in chemical validity were observed between the two representations:

DeepSMILES-based models yielded a higher fraction of syntactically valid predictions (54.2%) than SMILES-based models (43.5%). When considering only valid predictions, the two representations yield nearly identical performance (mean Tanimoto similarity of 0.67 for SMILES vs. 0.66 for DeepSMILES). However, this conditional metric does not capture the primary representation effect. The key difference lies in the frequency with which each representation produces a syntactically valid, scorable output. This is best reflected in the full test-set mean Tanimoto similarity (where invalid predictions are scored as 0), which we therefore adopt as the headline metric (Table 3).

Validity here reflects a strict conditional-recovery task (reproducing a specific reference fragment), and is not comparable to the high validity reported for unconditioned de novo generators. We note that invalid predictions are predominantly near-misses rather than noise: their mean string similarity to the reference is ∼0.60 (vs. ∼0.83 for valid predictions), indicating the models usually capture most of the target and fail on a local syntactic error, the failure mode DeepSMILES is designed to reduce. The hybrid raises validity to 65.3% because a valid prediction from either representation can be selected.

**Table 3:** Comparison of Tanimoto similarity, chemical validity, and Sequence Matcher ratio for SMILES, DeepSMILES, and combined predictions

Metric	SMILES	DeepSMILES	Combined
Mean Tanimoto Similarity	0.29 ± 0.42	0.36 ± 0.44	**0.43** ± **0.45**
Mean Chemical Validity (%)	43.5	54.2	**65.3**
Mean Sequence Matcher Ratio	**0.71**	0.61	0.66
Mean Tanimoto (Valid Predictions)	**0.67**	0.66	0.66
% Predictions with Tanimoto > 0.8	0.24	0.31	**0.39**

Bold indicates the highest value in each row

When evaluated on the test set, the mean Tanimoto similarity differed between representations as well, with DeepSMILES-based models yielding a mean value of 0.36 and SMILES-based models yielding a mean value of 0.29. Examination of the similarity distribution showed that 31% of DeepSMILES predictions and 24% of SMILES predictions exceeded a Tanimoto similarity of 0.8, suggesting that high-similarity predictions are distributed differently across the two representations rather than uniformly improved across all samples.

Paired comparisons of per-sample Tanimoto (two-tailed Wilcoxon signed-rank; n = 1,689; zeros dropped) with rank-biserial effect sizes, exact p-values, bootstrap 95% CIs, and a Holm correction are given in Table 4. A McNemar test on paired validity outcomes confirms DeepSMILES is valid significantly more often than SMILES: it is valid where SMILES fails in 369 cases versus the reverse in 188 (p = 1.4 × 10^–14^).

SMILES predictions achieved a higher mean string similarity (0.71) than DeepSMILES (0.61), while the hybrid was intermediate (0.66). SMILES-based models preserve greater character-level similarity, whereas DeepSMILES-based models more often produce predictions that are structurally closer at the molecular level, despite lower string-level similarity; the two metrics capture different aspects of prediction quality.

**Table 4:** Paired statistics on per-sample Tanimoto

Comparison	*Z*	*p*	Holm *p*	$r_{\textrm{rb}}$	mean Δ [95% CI]
DeepSMILES vs. SMILES	-6.998	$2.3\times 10^{-12}$	$2.3\times 10^{-12}$	+0.296	+0.066[+0.047,+0.086]
Hybrid vs. SMILES	-18.765	$3.9\times 10^{-79}$	$1.2\times 10^{-78}$	+1.000	+0.139[+0.124,+0.153]
Hybrid vs. DeepSMILES	-14.374	$5.6\times 10^{-47}$	$1.1\times 10^{-46}$	+1.000	+0.072[+0.062,+0.083]

Rank-biserial r=+1.000 for the hybrid rows is *definitional* (the oracle hybrid is never worse than a component); the substantive representation effect is the DeepSMILES-vs-SMILES row

As reported, drug-level overlap across train/test is low (1.6%), but fragment-level overlap is high (exact-fragment overlap 88.8%, Murcko-scaffold match 76.4%), reflecting the prevalence of frequently observed fragments. As direct evidence of the effect’s magnitude, we stratified test performance by reference-fragment frequency: on fragments that recur in the test set, mean Tanimoto is 0.41/0.48/0.58 (SMILES/DeepSMILES/hybrid), whereas on fragments appearing only once, it falls to 0.07/0.12/0.15; only 47% of reference fragments are distinct. Thus, the aggregate numbers are carried by frequently recurring fragments, and performance on rare (more plausibly novel) fragments is substantially weaker. The task, as constructed, primarily measures the model’s ability to contextualize known substructures rather than to generate entirely novel chemistry.

**Table 5:** Disjoint-fragment evaluation: mean Tanimoto on test fragments seen vs. unseen (exact canonical match) in the training set

Test subset	*n* (%)	SMILES	DeepSMILES	Hybrid
Overlapping (exact match in train)	1500 (88.8)	0.326	0.399	0.477
Novel (no exact match)	189 (11.2)	0.038	0.054	0.077
All test	1689 (100)	0.294	0.360	0.432

The relative ordering is preserved in both strata

As shown in Table 5, mean Tanimoto on seen fragments was 0.33, 0.40, and 0.48 for SMILES, DeepSMILES, and the hybrid model, respectively, whereas performance on novel fragments dropped substantially to 0.04, 0.05, and 0.08. The model, therefore, largely recalls training fragments and, as currently trained, does not generalize to novel chemistry. Crucially, the relative ordering (DeepSMILES> SMILES, hybrid> both) is preserved in both strata: the overlap inflates absolute similarity for both representations approximately equally and does not overturn the representation comparison that is this paper’s central claim.

**Table 6:** Hybrid selection breakdown

Category	Count	%	Interpretation
SMILES wins	275	16.3	SMILES Tanimoto strictly higher
DeepSMILES wins	469	27.8	DeepSMILES Tanimoto strictly higher
Tie: both invalid	588	34.8	Both models failed – correlated failure
Tie: both same score	357	21.1	Both found equivalent fragments
Total ties	**945**	**56.0**	

Bold indicates the aggregate total of the tie categories (588 + 357 = 945)

Selecting the higher-Tanimoto prediction per molecule (oracle hybrid) raised the mean Tanimoto to 0.43, with 39% of selected predictions exceeding 0.8. Among decisive molecules, DeepSMILES wins more often than SMILES (469 vs. 275; Table 6), and the average gain when each representation wins is comparable, so the two are genuinely complementary rather than one contributing rare-but-large gains.

The oracle hybrid requires the reference fragment and is therefore an upper bound. We tested a deployable selector that, for each molecule, chooses the representation with the lower per-prediction loss (the model’s own confidence, logged at inference; no reference used). This recovers most of the oracle gain (Table 7): mean Tanimoto 0.427 vs. the oracle’s 0.432, i.e., 93% of the gain over the best single representation, agreeing with the oracle on 94% of molecules where the representations differ. The improvement over the best single model is significant (Wilcoxon Z = − 17.738, p< 10^–70^; rank-biserial + 0.95), and the residual gap to the oracle is small but significant (Z = − 5.841, p = 5×10^−9^), indicating headroom for a better selector. The effect is robust to confidence normalization across the two tokenizers: length-normalized loss recovers 92.6% of the gain (94% agreement), per-model percentile-rank 86.3% (93%), and even a conservative per-model z-score 64.5%. A decoding-time ensemble that merges token probabilities from both representations is a natural next step.

**Table 7:** Deployable loss-based selector vs. the oracle hybrid (full-set mean Tanimoto)

Strategy	Mean Tanimoto
SMILES only	0.294
DeepSMILES only	0.360
Loss-based selector (deployable)	0.427
Oracle hybrid (upper bound)	0.432

Furthermore, we examined two representative examples from the test set. For each parent molecule, we compared fragments generated by the SMILES and DeepSMILES models across multiple analytical dimensions: structural similarity to reference fragments, chemical space context via nearest-neighbor analysis, and scaffold preservation assessment (Supporting Information).

**Fig. 5:**
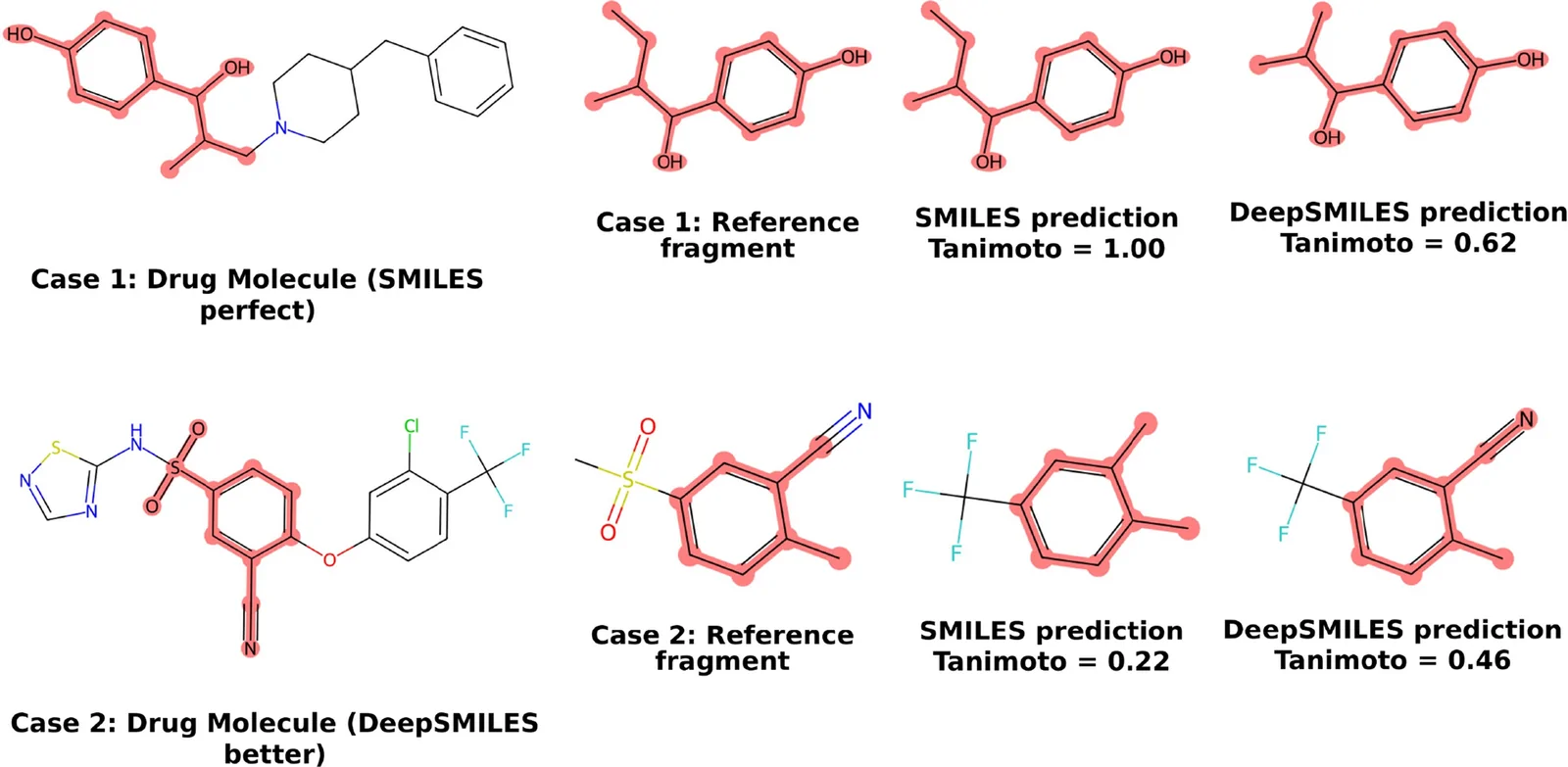
Top panel (Case 1): parent molecule with reference fragment highlighted, perfect SMILES prediction, and DeepSMILES alternative showing minor structural perturbations. Bottom panel (Case 2): parent molecule with sulfonamide–nitrile pharmacophore highlighted, incomplete SMILES prediction missing key groups, and corrected DeepSMILES prediction restoring critical binding elements

### Case 1: SMILES-dominated exact match

 In the first scenario, Fig.  5, the parent molecule contains a substituted phenyl–alcohol scaffold with clear fragmentation points. While the DeepSMILES component of the framework produces a structurally similar analogue (Tanimoto similarity = 0.62) with minor differences in the substitution pattern, the hybrid selection logic identifies the SMILES-based output, which achieves an exact recovery of the reference fragment (Tanimoto similarity = 1.00). This case highlights the framework’s ability to preserve exact matches by leveraging the syntactic precision of standard SMILES when appropriate.

### Case 2: DeepSMILES correction of key pharmacophoric elements

 Here, the framework compensates for the limitations of the SMILES-based model, which retains the aromatic ring and trifluoromethyl group but omits both the sulfonyl and nitrile groups present in the reference fragment (Tanimoto similarity = 0.22). The hybrid system utilizes the DeepSMILES component, which successfully recovers the nitrile group while preserving the aromatic core and trifluoromethyl substituent, improving structural relevance (Tanimoto similarity = 0.46). Although neither model fully captures the sulfonyl group, the DeepSMILES prediction restores a greater proportion of the reference pharmacophore. By selecting the DeepSMILES prediction, the framework preserves key pharmacophoric elements that would otherwise be lost in a single-representation system.

## Discussion

Our experiments underscored the importance of pretraining on a sufficiently large and relevant dataset [9, 20, 46]. DeepBERTa models without robust pretraining (e.g., starting from only 1k molecules) failed to learn the fragment task, essentially producing random fragments. Once we scaled pretraining to hundreds of thousands of molecules, performance markedly improved [11]. We suspect that both the choice of molecules—fragment-oriented or not—and the pretraining corpus can play a significant role in overall model performance [9, 11, 47].

In addition, the size of the fine-tuning dataset remains a fundamental limiting factor. With only 34,000 molecule–fragment pairs (all for the same target, mTOR), the model had a limited basis for generalization [5]. In a practical setting, one would ideally experiment on multiple targets to assess the generality of the results. Our focus on mTOR, while a useful case study, means the model may exploit idiosyncrasies of that particular protein. Notably, mTOR’s primary inhibitor, rapamycin, is a large, non-traditional drug molecule. Testing the method on more “drug-like” targets in the future would help validate its generality.

In both SMILES and DeepSMILES, a key drawback is that a molecule can often be broken into multiple meaningful fragments, and the model has no way of knowing which fragment was optimal. Our choice of metric, Tanimoto similarity, was appropriate in most cases, but exceptions arose when high-performing fragments were structurally dissimilar to the top-ranked reference. It is possible that a model predicted the second- or third-best fragment, but because our methodology had no way of identifying this, the prediction was penalized as if it were irrelevant. In future work, we plan to compare predictions with the second- and third-best fragments to mitigate this issue.

We acknowledge that Tanimoto similarity is a strict metric that penalizes any mismatch. However, this strictness is necessary for establishing a baseline [30]. In future work, we might consider alternative metrics or manual evaluation to better assess the usefulness of the predictions. For instance, a prediction that recovers the correct scaffold but is missing a substituent is arguably a success in terms of identifying the critical part of the molecule, even if the Tanimoto score is not high [48].

### Comparison with other approaches

Beyond choosing a robust notation, there are other ways to enforce validity and robustness in fragment prediction. One approach is to use an encoder–decoder model that generates the fragment string from the molecule (rather than our single-model MLM approach) [10, 49, 50]. Another approach is constrained decoding: for SMILES, one can incorporate grammar rules during generation to prevent invalid outputs [51].

In terms of generative modeling, one could envision a fragment prediction approach that is more akin to a retrieval or classification problem: selecting the most likely fragment from a predefined library [52]. This would guarantee valid outputs (since all library fragments are valid) and could be easier for a model to learn (multi-class classification). However, it limits predictions to known fragments and cannot suggest novel ones [53]. Our free-form generation allows novel fragment suggestions, which could be valuable in discovering new scaffolds, albeit with the occasional risk of structurally implausible predictions [54].

### Future directions

This work opens several avenues for future improvement. First, expanding the training data—both pretraining and fine-tuning—would likely boost performance for both SMILES and DeepSMILES. A model like DeepBERTa, trained on tens of millions of molecules, is expected to capture a broader range of chemical patterns, which may further improve fragment prediction behavior. On the fine-tuning side, incorporating fragment data for multiple targets (not just mTOR) or utilizing semi-supervised approaches (e.g., treating any high-scoring fragment from docking as a training example) could be beneficial.

Moreover, retrosynthetic fragmentation, which pairs the entire molecule with its most anchoring fragment (without the full ligand context), can introduce additional hydrogen bonds or bare polar groups that increase binding affinity. These artifacts can amplify biases in the results. Additionally, separating the fragment can modify the orientation or binding pocket engagement, since separated fragments may attach to different sites.

We recognize that retrosynthetic fragmentation can occasionally yield fragments with additional hydrogen-bond donors or acceptors, potentially inflating their docking scores and, by extension, the apparent predictive performance of models trained on such data. However, this inflation would apply uniformly to all models because both SMILES- and DeepSMILES-based pipelines were trained and evaluated on the exact same fragment set. As a result, while the absolute Tanimoto similarities reported here may be slightly optimistic, the relative differences between representations—and the consistent improvement observed in the hybrid approach—remain valid indicators of model capability. A sensitivity analysis confirms that fragment selection is robust to the weight of the docking term and is not systematically biased toward polar or charged fragments. As is common in retrosynthetic fragmentation over finite drug datasets, the same fragment scaffolds frequently recur across structurally distinct drugs, resulting in overlap between the fragment vocabularies of the training and test splits [55].

This affects absolute Tanimoto values across both representations. The Tanimoto scores reported reflect the model’s ability and do not distort the relative comparison between SMILES and DeepSMILES. Overall, the conclusions from this study are more relevant from a machine-learning and representation-learning perspective, whereas their direct biophysical interpretability is limited by the assumptions of the fragmentation algorithm.

Future work incorporating interaction-preserving fragmentation will allow us to reassess absolute performance under more physically rigorous conditions and techniques. One notable example of such an approach is MedChemFrag [56], one of the few methods designed specifically for interaction-preserving fragmentation, which performs ligand fragmentation explicitly in the context of a protein–ligand complex, thereby preserving key binding interactions. In addition, Receptor-based Reverse Fragment-Based Drug Design (R-FBDD) [57] or Fragment Molecular Orbital methods (specifically used for protein–ligand complexes) [58] can reduce predispositions by beginning with the ligand docked to the receptor and properly assessing the separated fragment’s energy with respect to the whole ligand–receptor complex.

## Conclusions

We present a study of transformer-based fragment prediction comparing SMILES and DeepSMILES representations and examining their complementarity through a per-sample hybrid selection analysis. We introduce DeepBERTa, a ChemBERTa-like transformer pretrained on DeepSMILES, and evaluate it alongside a SMILES-based counterpart under matched experimental conditions. Our results show that representation choice meaningfully influences prediction behavior: DeepSMILES yields higher syntactic validity and higher aggregate similarity when invalid outputs are included, whereas SMILES more closely preserves character-level similarity to reference fragments. A *post hoc* selection of the higher-similarity prediction across representations increases the aggregate similarity beyond either representation alone, indicating complementary strengths across different molecular contexts. Taken together, these findings motivate further investigation of representation-aware modeling and selection strategies in cheminformatics.

## Supplementary Information

Supplementary material 1.

## Data Availability

All training, evaluation, and preprocessing code, as well as pretrained models, are available at https://github.com/AayushK-othari/FBDD_ChemBERTa and on Hugging Face under the username *aakothari*. The datasets used for model training and validation are derived from publicly available ZINC20 resources. Selection and preprocessing scripts are included in the repository to ensure reproducibility.

## Abbreviations

FBDD: Fragment-based drug discovery
SMILES: Simplified Molecular Input Line Entry System
DeepSMILES: Deep Simplified Molecular Input Line Entry System
MLM: Masked language modeling
MTR: Multitask regression
NLP: Natural language processing
RECAP: Retrosynthetic Combinatorial Analysis Procedure
HTS: High-throughput screening
CE: Cross-entropy
BPE: Byte-pair encoding
SELFIES: Self-Referencing Embedded Strings
RNN: Recurrent neural network
VAE: Variational autoencoder
mTOR: Mechanistic target of rapamycin
Da: Dalton
GELU: Gaussian Error Linear Unit
ESOL: Estimated Solubility
SAscore: Synthetic Accessibility score
TPE: Tree-structured Parzen Estimator
R-FBDD: Receptor-based Reverse Fragment-Based Drug Design
LLM: Large Language Model

## References

[1] Murray CW, Rees DC (2009) The rise of fragment-based drug discovery. Nat Chem 1(3):187–192 10.1038/nchem.217 21378847

[2] Erlanson DA, Fesik SW, Hubbard RE, Jahnke W, Jhoti H (2016) Twenty years on: the impact of fragments on drug discovery. Nat Rev Drug Discovery 15(9):605–619 10.1038/nrd.2016.109 27417849

[3] Schneider G, Clark DE (2019) Automated de novo drug design: Are we nearly there yet? Angew Chem 131(32):10906–10917 10.1002/anie.201814681 30730601

[4] Lo Y-C, Rensi SE, Torng W, Altman RB (2018) Machine learning in chemoinformatics and drug discovery. Drug Discovery Today 23(8):1538–1546 10.1016/j.drudis.2018.05.010 PMC6078794 29750902

[5] Jin W, Barzilay R, Jaakkola T (2018) Junction tree variational autoencoder for molecular graph generation. arXiv:1802.04364 arXiv preprint

[6] Weininger D (1988) Smiles, a chemical language and information system. 1. Introduction to methodology and encoding rules. J Chem Inf Comput Sci 28(1):31–36

[7] Kim S, Thiessen PA, Bolton EE, Chen J, Fu G, Gindulyte A, Han L, He J, He S, Shoemaker BA, Wang J, Yu B, Zhang J, Bryant SH (2015) Pubchem substance and compound databases. Nucleic Acids Res 44(D1):1202–1213 10.1093/nar/gkv951 PMC4702940 26400175

[8] Sterling T, Irwin JJ (2015) Zinc 15—ligand discovery for everyone. J Chem Inf Model 55(11):2324–2337 10.1021/acs.jcim.5b00559 PMC4658288 26479676

[9] Chithrananda S, Grand G, Ramsundar B (2020) Chemberta: large-scale self-supervised pretraining for molecular property prediction. arXiv:2010.09885 arXiv preprint

[10] Schwaller P, Laino T, Gaudin T, Bolgar P, Hunter CA, Bekas C, Lee AA (2019) Molecular transformer: a model for uncertainty-calibrated chemical reaction prediction. ACS Cent Sci 5(9):1572–1583 10.1021/acscentsci.9b00576 PMC6764164 31572784

[11] Wang S, Guo Y, Wang Y, Sun H, Huang J (2019) Smiles-BERT. In: Proceedings of the 10th ACM international conference on bioinformatics, computational biology and health informatics, New York, NY, USA, pp. 429–436. ACM. [Online; accessed 2025-07-31]

[12] Maziarka D, Mucha T, Rataj S, Tabor K, Jastrzebski J, S (2020) Molecule attention transformer. arXiv:2002.08264 arXiv preprint

[13] Vaswani A, Shazeer N, Parmar N, Uszkoreit J, Jones L, Gomez AN, Kaiser Polosukhin I (2017) Attention is all you need. arXiv preprint arXiv:1706.03762

[14] Ahmad W, Simon E, Chithrananda S, Grand G, Ramsundar B (2022) Chemberta-2: towards chemical foundation models. arXiv preprint arXiv:2209.01712

[15] Honda S, Shi S, Ueda H (2019) Smiles transformer: Pre-trained molecular fingerprint for low data drug discovery. arXiv preprint arXiv:1911.04738

[16] Fabian B, Edlich T, Gaspar H, Segler M, Meyers J, Fiscato M, Ahmed M (2020) BenevolentAI: molecular representation learning with language models and domain-relevant auxiliary tasks. arXiv preprint arXiv:2011.13230

[17] Bagal V, Aggarwal R, Vinod PK, Priyakumar UD (2021) Molgpt: molecular generation using a transformer-decoder model. J Chem Inf Model 62(9):2064–2076 10.1021/acs.jcim.1c00600 34694798

[18] Ross J, Belgodere B, Hoffman SC, Chenthamarakshan V, Navratil J, Mroueh Y, Das P (2024) Gp-molformer: a foundation model for molecular generation. arXiv preprint arXiv:2405.04912

[19] Singh R, Barsainyan AA, Irfan R, Amorin CJ, He S, Davis T, Thiagarajan A, Sankaran S, Chithrananda S, Ahmad W, Jones D, McLoughlin K, Kim H, Bhutani A, Sathyanarayana SV, Viswanathan V, Allen JE, Ramsundar B (2025) Chemberta-3: an open source training framework for chemical foundation models. Technical report, American Chemical Society (ACS). [Online; accessed 2025-07-31]

[20] Rong Y, Bian Y, Xu T, Xie W, Wei Y, Huang W, Huang J (2020) Self-supervised graph transformer on large-scale molecular data. arXiv preprint arXiv:2007.02835

[21] Ucak UV, Ashyrmamatov I, Lee J (2023) Improving the quality of chemical language model outcomes with atom-in-SMILES tokenization. J Cheminform. 10.1186/s13321-023-00725-9 PMC10228139 37248531

[22] Krenn M, Häse F, Nigam A, Friederich P, Aspuru-Guzik A (2020) Self-referencing embedded strings (SELFIES): a 100% robust molecular string representation. Mach Learn Sci Technol 1(4):045024

[23] Kusner MJ, Paige B, Hernández-Lobato JM (2017) Grammar variational autoencoder. arXiv preprint arXiv:1703.01925

[24] O’Boyle N, Dalke A (2018) Deepsmiles: an adaptation of SMILES for use in machine-learning of chemical structures. Technical report, American Chemical Society (ACS). [Online; accessed 2025-07-31]

[25] Berenger F, Tsuda K (2021) Molecular generation by fast assembly of (Deep)SMILES fragments. J Cheminform 13(1):27 10.1186/s13321-021-00566-4 PMC8591910 34775976

[26] Hajduk PJ, Huth JR, Fesik SW (2005) Druggability indices for protein targets derived from NMR-based screening data. J Med Chem 48(7):2518–2525 10.1021/jm049131r 15801841

[27] Lewell XQ, Judd DB, Watson SP, Hann MM (1998) Cheminform abstract: recap – retrosynthetic combinatorial analysis procedure: a powerful new technique for identifying privileged molecular fragments with useful applications in combinatorial chemistry. ChemInform. 10.1002/chin.199836303 9611787

[28] Erlanson DA, Wells JA, Braisted AC (2004) Tethering: fragment-based drug discovery. Annu Rev Biophys Biomol Struct 33(1):199–223 10.1146/annurev.biophys.33.110502.140409 15139811

[29] Ortega-Ochoa R, Vegge T, Frellsen J (2024) Molminer: towards controllable, 3d-aware, fragment-based molecular design. arXiv preprint arXiv:2411.06608

[30] Nigam A, Pollice R, Krenn M, Gomes GDP, Aspuru-Guzik A (2021) Beyond generative models: superfast traversal, optimization, novelty, exploration and discovery (STONED) algorithm for molecules using SELFIES. Chem Sci 12(20):7079–7090 10.1039/d1sc00231g PMC8153210 34123336

[31] Brown N, Fiscato M, Segler MHS, Vaucher AC (2019) Guacamol: benchmarking models for de novo molecular design. J Chem Inf Model 59(3):1096–1108 10.1021/acs.jcim.8b00839 30887799

[32] Skinnider MA (2024) Invalid smiles are beneficial rather than detrimental to chemical language models. Nat Mach Intell 6(4):437–448

[33] Xu D, Pan Q, Yuan S, Li J, Zhu Z, Ji J (2026) Unveiling scaling behaviors in molecular language models: effects of model size, data, and representation. arXiv:2601.22757 arXiv preprint

[34] Congreve M, Carr R, Murray C, Jhoti H (2003) A rule of three for fragment-based lead discovery? Drug Discov Today 8(19):876–877 10.1016/s1359-6446(03)02831-9 14554012

[35] Delaney JS (2004) Esol: estimating aqueous solubility directly from molecular structure. J Chem Inf Comput Sci 44(3):1000–1005 10.1021/ci034243x 15154768

[36] Ertl P, Schuffenhauer A (2009) Estimation of synthetic accessibility score of drug-like molecules based on molecular complexity and fragment contributions. J Cheminform 1(1):8 10.1186/1758-2946-1-8 PMC3225829 20298526

[37] Liu Y, Ott M, Goyal N, Du J, Joshi M, Chen D, Levy O, Lewis M, Zettlemoyer L, Stoyanov V (2019) Roberta: a robustly optimized Bert pretraining approach. arXiv preprint arXiv:1907.11692

[38] Bajusz D, Rácz A, Héberger K (2015) Why is Tanimoto index an appropriate choice for fingerprint-based similarity calculations. J Cheminform. 10.1186/s13321-015-0069-3 PMC4456712 26052348

[39] Rogers D, Hahn M (2010) Extended-connectivity fingerprints. J Chem Inf Model 50(5):742–754 10.1021/ci100050t 20426451

[40] Loshchilov I, Hutter F (2017) Decoupled weight decay regularization. arXiv preprint arXiv:1711.05101. [Online; accessed 2025-07-31]

[41] Devlin J, Chang M-W, Lee K, Toutanova K (2019) BERT: Pre-training of deep bidirectional transformers for language understanding. In: Burstein J, Doran C, Solorio T (eds) Proceedings of the 2019 conference of the north American chapter of the association for computational linguistics: human language technologies, Volume 1 (Long and Short Papers). Association for Computational Linguistics, Minneapolis, Minnesota, pp 4171–4186. 10.18653/v1/N19-1423

[42] Landrum G (2006) RDKit: open-source cheminformatics. http://www.rdkit.org

[43] Akiba T, Sano S, Yanase T, Ohta T, Koyama M (2019) Optuna: a next-generation hyperparameter optimization framework. Proceedings of the 25th ACM SIGKDD international conference on knowledge discovery and data mining

[44] Rey D, Neuhäuser M (2011) Wilcoxon-signed-rank test. International encyclopedia of statistical science. pp 1658–1659

[45] Foundation PS (2024) difflib—Helpers for computing deltas. https://docs.python.org/3/library/difflib.html. [Online; accessed 2025-08-03]

[46] Qiao J, Gao W, Jin J, Wang D, Guo X, Manavalan B, Wei L (2025) Molecular pretraining models towards molecular property prediction. Sci China Inf Sci. 10.1007/s11432-024-4457-2

[47] Winter R, Montanari F, Noé F, Clevert D-A (2019) Learning continuous and data-driven molecular descriptors by translating equivalent chemical representations. Chem Sci 10(6):1692–1701 10.1039/c8sc04175j PMC6368215 30842833

[48] Jiménez-Luna J, Grisoni F, Schneider G (2020) Drug discovery with explainable artificial intelligence. Nat Mach Intell 2(10):573–584

[49] Kotsias P-C, Arús-Pous J, Chen H, Engkvist O, Tyrchan C, Bjerrum EJ (2020) Direct steering of de novo molecular generation with descriptor conditional recurrent neural networks. Nat Mach Intell 2(5):254–265

[50] Soares E, Vital Brazil E, Shirasuna V, Zubarev D, Cerqueira R, Schmidt K (2025) An open-source family of large encoder-decoder foundation models for chemistry. Commun Chem 8(1):27 10.1038/s42004-025-01585-0 PMC12216393 40593316

[51] Geng S, Josifoski M, Peyrard M, West R (2023) Grammar-constrained decoding for structured nlp tasks without finetuning. In: Proceedings of the 2023 Conference on empirical methods in natural language processing, Stroudsburg, PA, USA, pp. 8122–8144. Association for Computational Linguistics. https://aclanthology.org/2023.emnlp-main.508

[52] Jia Q, Zhang Y, Wang Y, Ruan T, Yao M, Wang L (2025) Fragment-level feature fusion method using retrosynthetic fragmentation algorithm for molecular property prediction. J Mol Graph Model 137:108985 10.1016/j.jmgm.2025.108985 40009893

[53] Green H, Koes DR, Durrant JD (2021) Deepfrag: a deep convolutional neural network for fragment-based lead optimization. Chem Sci 12(23):8036–8047 10.1039/d1sc00163a PMC8208308 34194693

[54] Thomas M, Ahmad M, Tresadern G, Fabritiis G (2024) Promptsmiles: Prompting for scaffold decoration and fragment linking in chemical language models. Technical report, American Chemical Society (ACS). [Online; accessed 2025-07-31] 10.1186/s13321-024-00866-5 PMC11225391 38965600

[55] Chávez-Hernández AL, Sánchez-Cruz N, Medina-Franco JL (2020) Fragment library of natural products and compound databases for drug discovery. Biomolecules 10(11):1518 10.3390/biom10111518 PMC7694623 33172012

[56] Ivanov NN, Shulga DA, Palyulin VA (2023) Decomposition of small molecules for fragment-based drug design. Biophysica 3(2):362–372

[57] Shulga DA, Ivanov NN, Palyulin VA (2021) Reverse fragment based drug discovery approach via simple estimation of fragment contributions. Mendeleev Commun 31(3):291–293. 10.1016/j.mencom.2021.04.004

[58] Kitaura K, Ikeo E, Asada T, Nakano T, Uebayasi M (1999) Fragment molecular orbital method: an approximate computational method for large molecules. Chem Phys Lett 313(3–4):701–706. 10.1016/S0009-2614(99)00874-X

