# Supplementary material for: Transformer-based molecular fragment prediction using SMILES and DeepSMILES representations in a fragment-based drug discovery pipeline
Source: J Cheminform. 2026 Jul 31;18:148. doi: 10.1186/s13321-026-01255-w (PMC13721515; doi:10.1186/s13321-026-01255-w)
Supplement: Supplementary file 1 [file 13321_2026_1255_MOESM1_ESM.pdf]

# Transformer-Based Molecular Fragment Prediction Using SMILES and DeepSMILES Representations in a Fragment-Based Drug Discovery Pipeline

Aayush Kothari,[†] Amish Gupta,[†] Nisarg Shah,[‡] Thomas Reed,[‡] Anvita Nattuva,[‡] Rahima Nazirudeen,[‡] Harman Brah,[‡] and Marx Akl[*,†]

†*Department of Computer Science and Engineering, Aspiring Scholars Directed Research Program, Fremont, CA 94539, United States*

‡*Department of Chemistry, Biochemistry, & Physical Science, Aspiring Scholars Directed Research Program, Fremont, CA 94539, United States*

E-mail: marx.akl@asdrp.org

## Table of Contents (Supporting Information)

- **Section S1.** Data & Code Availability
- **Section S2.** Dataset Details
  - S2.1 Dataset Source
  - S2.2 Preprocessing and Cleaning
  - S2.3 Fragment Generation (RECAP)
  - S2.4 Docking Procedure

  - S2.5 Final Dataset and Splits
  - S2.6 Software
  - S2.7 Availability (GitHub/Hugging Face)
- **Section S3.** DeepBERTa Training Configuration
- **Section S4.** Molecular Case Study Results


## Data & Code Availability

All data, code, and model artifacts for this study are openly available:

- **GitHub (code + environment files):** end-to-end preprocessing, training, and evaluation pipelines, with `environment.yml`/`requirements.txt`, reproducible scripts, and figure code.

  *Link:* `https://github.com/AayushK-othari/FBDD-ChemBERTa`

- **Hugging Face (models + datasets):** pretrained/finetuned checkpoints (SMILES, DeepSMILES, DeepBERTa) and tokenizer artifacts. *Link:* `https://huggingface.co/aakothari`

- **Weights & Biases (modeling specifics/logs for DeepBERTa):** experiment dashboards, per-seed runs, hyperparameters, losses, and evaluation artifacts.

  *Project:* `https://wandb.ai/aakothari/huggingface`

Where applicable, we recommend archiving releases with persistent DOIs (e.g., Zenodo) upon acceptance; links will be updated accordingly.

# Dataset Details

## Dataset Source

The initial dataset of drugs was obtained from a mix of ChemBL and PubChem. This dataset contained approximately 34,000 molecules in SMILES format.

## Preprocessing and Cleaning

Prior to use in our pipeline, we performed several preprocessing steps to ensure quality and consistency:

- **Duplicate removal:** all repeated SMILES entries were removed. Duplicate rows as well as molecules with more than one best fragment were removed.
- **Canonicalization:** all SMILES were canonicalized using RDKit to ensure uniqueness and consistency.
- **DeepSMILES conversion:** molecules were converted into DeepSMILES notation using the O'Boyle and Dalke converter.

## Fragment Generation

Molecules were fragmented using the **RECAP algorithm** as implemented in RDKit. We collected the terminal (leaf) fragments produced by the decomposition. To enable docking, any wildcard atoms at cut points were replaced with carbon atoms, and fragments were geometry-optimized (RDKit: hydrogen addition, 3D embedding with a fixed random seed, UFF optimization, hydrogen removal).

## Docking Procedure

Protein preparation was performed by converting the supplied `.pdb` structure to `.pdbqt` using AutoDockTools 1.5.7, which was also used to obtain the docking box center and dimensions.

Docking was performed using Uni-Dock to speed up data generation. See `gen_data_main.py` for dataset generation and `rank_fragments.py` for the bulk fragment scoring function. Due to issues when running Uni-Dock on very large sets of ligands, `split_datasets.py` is used to split the drugs input dataset and write a batch file of commands to generate training dataset chunks which are then merged with `combine_dataset_chunks.py`. All final SMILES strings were stripped of stereochemistry and canonicalized. As a workaround to unpredictable crashes with Uni-Dock when given large numbers of ligands, we broke up our input dataset into chunks and then consolidated the output chunks. This implementation detail does not affect our results.

## Final Dataset

After cleaning, fragmentation, and docking, the dataset consisted of approximately **34,000 unique drug–fragment pairs**. The dataset was split into:

- Training: 80%
- Validation: 15%
- Test: 5%

The same splits were used for SMILES and DeepSMILES pipelines to ensure comparability.

## Software

All experiments involving SMILES and DeepSMILES for downstream tasks were conducted on an HP ZBook Fury G8 Mobile Workstation, equipped with an NVIDIA RTX A3000 Laptop GPU, an Intel Core i7-11850H processor (8 cores/16 threads, 2.50 GHz), 32 GB of RAM, and running Windows 11. DeepBERTa pretraining was conducted using 3 NVIDIA Tesla P100-PCIE-12GB GPUs.

## Availability

The processed dataset (cleaned SMILES, DeepSMILES, and selected fragments) is openly available at:

- GitHub: `https://github.com/AayushK-othari/FBDD-ChemBERTa`
- Hugging Face: `https://huggingface.co/aakothari`

# DeepBERTa Training Configuration and Results

Table S1: Training hyperparameters for DeepBERTa-v4.

| Parameter | Value |
|---|---|
| Learning rate | $5.0 \times 10^{-5}$ (linear decay) |
| Batch size | 8 |
| Optimizer | AdamW |
| Epochs trained | 0.35 |
| Gradient clipping | None (default) |
| Random seed | 42 |

Table S2: Training and evaluation metrics for DeepBERTa-v4.

| Metric | Value |
|---|---|
| Final training loss | 1.43 |
| Final evaluation loss | 1.58 |
| Training runtime | 3253 s (54 min) |
| Training throughput | 292 samples/s, 146 steps/s |
| Evaluation runtime | $\sim$55 s per eval |
| Evaluation throughput | 91 samples/s |
| Learning rate range | $5.0 \times 10^{-5} \rightarrow 4.8 \times 10^{-5}$ |
| Final gradient norm | 9.37 |

# Molecular Case Study Results

Table S3: Expanded hyperparameter configuration for DeepBERTa.

| **Parameter** | **Value** |
|---|---|
| Tokenizer | DeepSMILES (vocab size = 600) |
| Max sequence length | 128 |
| Hidden size | 768 |
| Number of layers | 6 |
| Attention heads | 12 |
| Dropout | 0.1 |
| Activation | GELU |
| Optimizer | AdamW |
| Learning rate schedule | Linear decay with warmup |
| Initial learning rate | $5.0 \times 10^{-5}$ |
| Warmup steps | 1000 |
| Batch size | 8 |
| Epochs trained | 0.35 |
| Gradient clipping | None |
| Weight decay | 0.01 |
| Random seed | 42 |

Table S4: Comparison of physicochemical and structural metrics for representative fragment prediction cases. Functional group score denotes the degree of functional group preservation relative to the reference fragment (1.0 = perfect match).

| **Metric** | **Case 1 Ref** | **Case 1 Hybrid** | **Case 2 Ref** | **Case 2 Hybrid** | **Case 3 Ref** | **Case 3 Hybrid** |
|---|---|---|---|---|---|---|
| Molecular Weight | 164.20 | 164.20 | 176.17 | 176.17 | 245.33 | 243.32 |
| Heavy Atoms | 12 | 12 | 13 | 13 | 18 | 18 |
| Aromatic Rings | 1 | 1 | 1 | 1 | 2 | 2 |
| H-Bond Donors | 2 | 2 | 1 | 1 | 1 | 1 |
| H-Bond Acceptors | 2 | 2 | 4 | 4 | 3 | 3 |
| Rotatable Bonds | 3 | 3 | 2 | 2 | 4 | 4 |
| Murcko Scaffold Match | – | Exact | – | Exact | – | Partial (core preserved) |
| Functional Group Score | – | 1.00 | – | 0.83 | – | 0.78 |
